# Using Ambulatory Assessment to Explore Links between Daily Activities and Daily life Cognitive Functioning among Older Adults

**DOI:** 10.1002/alz.090464

**Published:** 2025-01-03

**Authors:** Andrea M. Weinstein, Scott Rothenberger, Raeanne C Moore, Beth E. Snitz, Ann D Cohen, Meryl A Butters

**Affiliations:** ^1^ University of Pittsburgh, Pittsburgh, PA USA; ^2^ University of California San Diego, La Jolla, CA USA; ^3^ School of Medicine, University of Pittsburgh, Pittsburgh, PA USA; ^4^ University of Pittsburgh Alzheimer’s Disease Research Center (ADRC), Pittsburgh, PA USA

## Abstract

**Background:**

Characterizing dementia risk is difficult with traditional neuropsychological testing. Ecological Momentary Cognitive Testing (EMCT) uses ecological momentary assessment (EMA) methods to sample daily life cognition. The current study examined how daily activities were related to EMCT performance and explored whether associations remained when stratifying by amyloid‐beta (Aβ) status.

**Method:**

Older adults without dementia underwent Pib‐PET imaging and a 10‐day EMA and EMCT protocol using the NeuroUX platform. EMCT measures included verbal memory (12‐item Memory List accuracy), spatial memory (Memory Matrix accuracy), inhibitory control (Color Trick reaction time [RT]), and visual search/processing speed (Matching Pair RT). We examined EMCT in relation to concurrent and previous day activities reported by EMA: 1) work/volunteer activities and 2) physical leisure activities (e.g., exercising or gardening). EMCT task performance was calculated by intraindividual mean and variability, assuming higher mean accuracy and lower variability indicated better cognition. We jointly modeled the mean function and log transformed intraindividual variability for each EMCT task in relation to concurrent and previous day activity, controlling for age, education, and protocol day. We then explored associations stratified by Aβ status.

**Result:**

Forty participants (age = 73.6 years [67‐91]; education = 15 years [10‐19]; 21 female; 77% white; 7 Aβ+) completed the protocol. For instance, concurrent physical leisure (*β* = ‐.15, p = .011) or work activities *(β = ‐.15*, p = .012) was associated with lower Color Trick RT variability and this association held in Aβ+ individuals *(β = ‐.69*, p<.01; *β* = ‐.39, p = .01). Concurrent physical activities were associated with higher Memory List mean accuracy *(β = .67*, p = .05) and concurrent work activities was associated with lower Memory List variability (b = ‐.15, p = .04). Conversely, concurrent physical leisure activities were associated with higher Matching Pair RT variability *(β = .15*, p = .02) but only in Aβ‐ individuals. When looking at previous day activities, physical leisure *(β = ‐.13*, p = .05) and work *(β = ‐.16*, p = .02) activities were associated with less next‐day Memory Matrix variability, in both Aβ+ and Aβ‐ individuals.

**Conclusion:**

Daily cognitive performance (i.e., EMCT) is sensitive to daily activities, with possible differences by Aβ status. Ongoing work is further examining these patterns (K23AG076663).

